# Case Report: Transcatheter Arterial Embolization for the Initial Management of Intra-Abdominal Hemorrhage From a Hepatic Tumor in a Cat

**DOI:** 10.3389/fvets.2021.707120

**Published:** 2021-08-02

**Authors:** Yuta Kawamura, Hiroki Itou, Akitomo Kida, Hiroki Sunkawa, Kenji Kawamura

**Affiliations:** ^1^Kawamura Animal Hospital, Niigata, Japan; ^2^Yamagata University Hospital, Yamagata, Japan

**Keywords:** transcatheter arterial embolization, intra-abdominal hemorrhage, hemostasis, hepatic mass, feline

## Abstract

An 8-year-old Ragdoll cat was admitted to our hospital after its owner noticed sudden lethargy. Abdominal ultrasonography showed a large amount of blood in the abdominal cavity, and the cat was diagnosed as having hemorrhagic shock caused by the rupture of an intra-abdominal mass. Blood transfusion was performed on the 1st day of hospitalization. On the 2nd day, contrast-enhanced computed tomography (CT) was performed, and hemorrhage from a mass originating in the caudate lobe of the liver was noted. Transcatheter arterial embolization (TAE) was performed to stop the bleeding from the mass using Gelpart to embolize the feeding artery. The following day, fever and elevation of liver enzyme levels were observed, but these subsided within a few days. At discharge 5 days after TAE, no fluid was found in the peritoneal cavity, and no further intra-abdominal bleeding occurred. Sixty-six days after TAE, we were able to perform resection surgery with the cat in good condition. A partial response was observed on CT performed before surgery. Histopathology revealed cholangiocellular adenoma. The cat was doing well as of postoperative day 549. This case indicates that TAE may be effective for initial hemostasis and stabilization of conditions in animals with tumor-induced hemorrhage.

## Introduction

Non-traumatic intra-abdominal hemorrhage often results from the rupture of a neoplasm, such as in the spleen or liver, leading to a reduction in a patient's circulating blood volume and shock; therefore, rapid and appropriate treatment is important. In veterinary medicine, surgery for hemostasis is often performed after stabilizing the patient's condition, but the prognosis is poor. In contrast, in human medicine, transcatheter arterial embolization (TAE) is often used as the first-choice treatment to achieve rapid hemostasis and better prognosis. In this study, TAE was performed for hemostasis in a cat with hemorrhagic shock caused by the rupture of a liver tumor.

## Case Description

An 8-year-old neutered male cat weighing 4.9 kg was brought to Kawamura Animal Hospital for a sudden loss of energy and inappetence that developed on the same morning.

Physical examination at the first visit revealed a body condition score of 3/5, body temperature of 38.3°C, heart rate of 216 beats/min, tachypnea, visible pallor, and capillary refill time of 2.0 s.

Based on the above physical examination findings, hypovolemic shock, cardiogenic shock, obstructive shock, respiratory diseases, etc. were suspected, and blood examination, chest and abdominal radiological examination, and abdominal ultrasound examination were performed.

Blood test results revealed severe anemia [packed cell volume (PCV), 16%; reference range, 29–48%] but no other major abnormalities.

There were no obvious abnormalities in the chest on radiological examination. However, the presence of a mass in the upper abdominal region was suspected, and a decrease in serosal detail was observed on abdominal radiographs.

Abdominal ultrasonography revealed a 40-mm-sized mass in the right upper quadrant and fluid accumulation in the abdominal cavity. The fluid was aspirated, and blood was found on analysis of the aspirate. This finding along with the cat's symptoms suggested shock owing to sudden bleeding from the intra-abdominal mass.

## Diagnostic Assessment, Therapeutic Intervention, and Outcomes

### Treatment and Clinical Course

**Figure 1:**
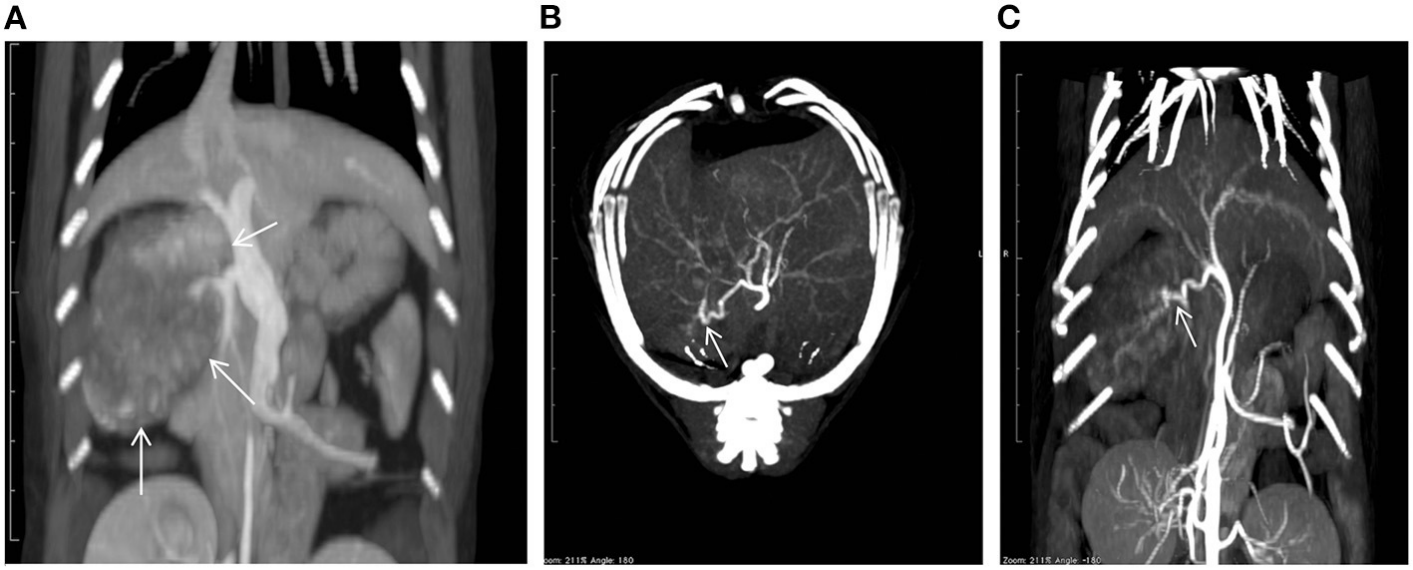
A computed tomography (CT) image showing a mass (arrows) in the caudate lobe of the liver **(A)**. Maximum intensity projection CT images taken in the arterial phase [**(B)**: axial; **(C)**: coronal] showing the feeding vessels of the tumor (arrows).

**Figure 2:**
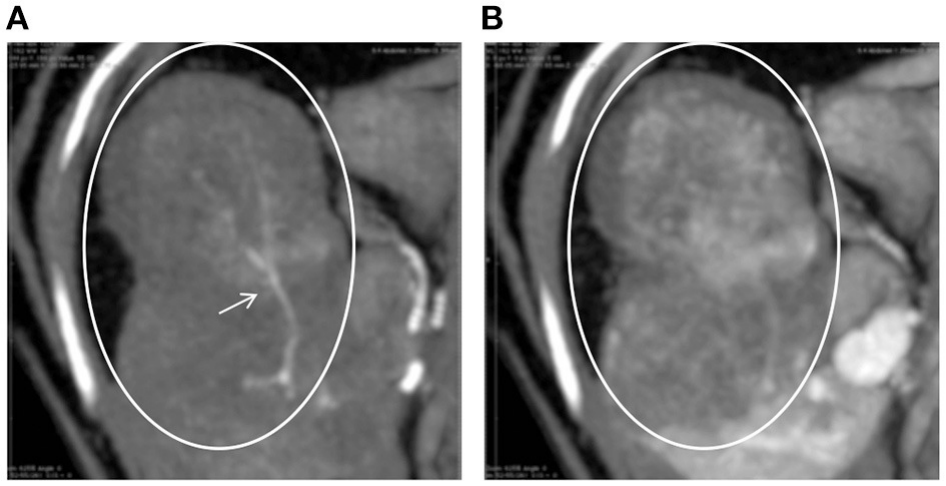
CT images of the hepatic tumor (circles) and surrounding liver parenchyma in the arterial **(A)** and portal **(B)** phases. Tumor vessels (arrow) are clearly visible only in the arterial phase. In the portal phase, the tumor area shows a lower CT value than the surrounding areas.

On the first day of hospitalization, oxygenation in an oxygen cage with 30% oxygen concentration and blood transfusion (100 mL of whole blood expected to increase PCV approximately 10%) were performed to stabilize the cat's condition. On day two, contrast-enhanced computed tomography (CT) was performed. The cat was premedicated with atropine sulfate (0.01 mg/kg; intravenous [IV]), midazolam (0.25 mg/kg; IV), and fentanyl (5 μg/kg; IV bolus). Intravenous propofol (3.0 mg/kg) was used to induce anesthesia. An endotracheal tube (ID 4.0 mm) was placed, and anesthesia was maintained with isoflurane (1.6–2.0%) in oxygen. Ephedrine hydrochloride (1 mg/kg; IV bolus, as necessary) was used to maintain blood pressure. For CT examination, a 16-row multi-slice CT scanner (Brivo CT385, GE Medical Systems, Chicago, IL) was used. The cat was administered 2.0 mL/kg iohexol contrast medium (300 mgI/mL) (Omnipaque 300 injection, GE Healthcare Pharma, Chicago, IL), which was injected intravenously at 0.25 mL/s. Images were acquired during the arterial phase (20 s), portal phase (40 s), and equilibrium phase (120 s) (slice thickness: 1.2 mm). An intra-abdominal mass originating from the caudate lobe of the liver was confirmed (Figure 1A). Additionally, severe inflammation and accumulation of bloody ascites were noted at the periphery of the mass. In the arterial phase, the caudate branch of the hepatic artery was identified as the main vessel feeding the mass (Figures 1B,C). The diameter of the main artery that fed the tumor was 1.2 mm, and the diameter of the first branch was 0.6 mm. In the portal vein phase, lower CT values were observed for the liver mass (90–100 HU) than for the normal liver parenchyma (210–220 HU), and portal blood flow was not confirmed in the liver mass (Figure 2). These findings suggested that the mass was mainly supplied by the surrounding arteries rather than by the portal vein. In human medicine, these findings also indicate that the tumor receives blood from the arteries rather than from the portal vein (1, 2). Fine needle aspiration cytology of the mass showed hepatocyte-like cells.

After consultation with the owner, TAE was scheduled after CT examination to reduce hemorrhage from the tumor.

### TAE Procedure and Findings

Cefovecin sodium (8 mg/kg) was administered subcutaneously to the cat as a perioperative antibiotic. The cat was held in the supine position, and the right groin was shaved and disinfected.

**Figure 3:**
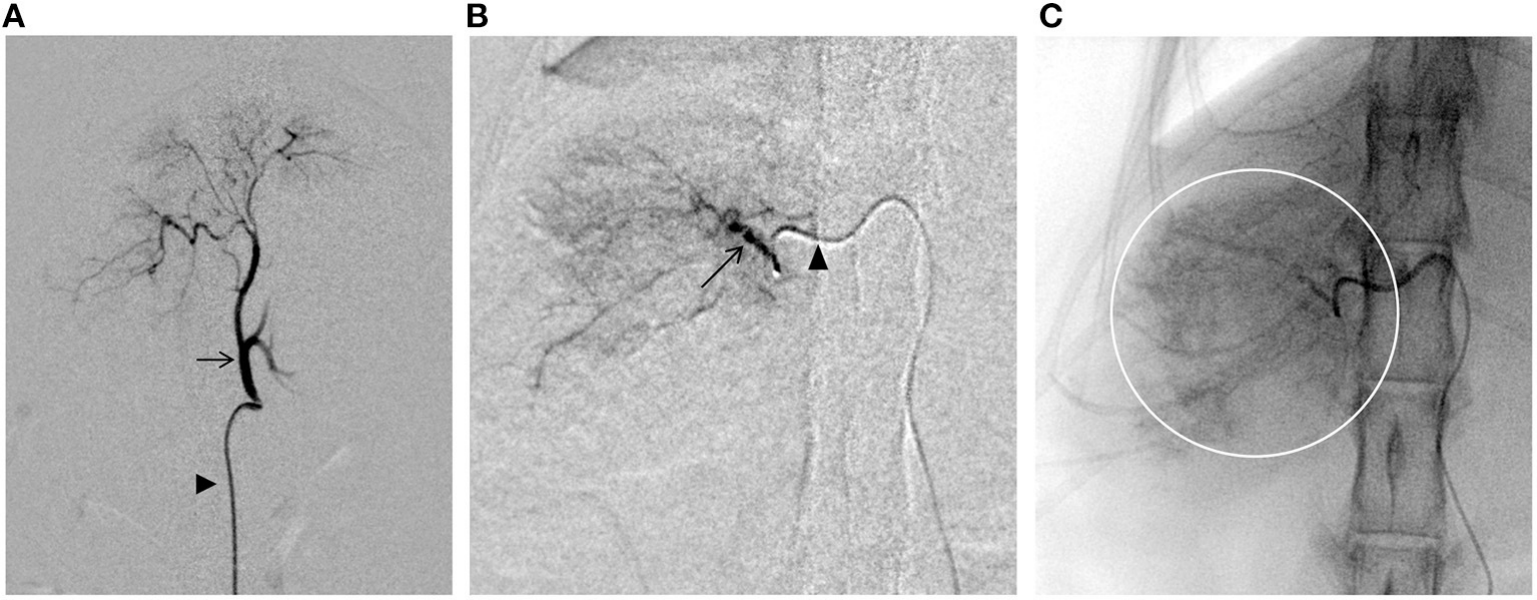
Fluoroscopic images obtained during the transcatheter arterial embolization (TAE) procedure. **(A)** Angiography of the celiac artery (arrows). Arrowhead indicates the 4.2-Fr RIM-S catheter. **(B)** Angiography of the nutritional vessels (arrows). Arrowhead indicates the 1.7-Fr microcatheter. **(C)** Embolization of the tumor through the caudate branch of the common hepatic artery. The presence of contrast-enhanced emboli can be seen throughout the tumor area (circle).

The following procedure was performed using a flat-panel-type X-ray fluoroscope (CIOS fusion, Siemens, Munich, Germany). A 4-Fr short sheath (Radifocus Introducer IIH, Terumo, Tokyo, Japan) was inserted into the right femoral artery using a cut-down technique. A 4.2-Fr RIM-S guiding catheter (4.2-Fr RIM-S guiding catheter, Hanaco Medical, Saitama, Japan) was then inserted. The catheter was advanced beyond the celiac artery in the abdominal aorta and the celiac artery was isolated by slowly pulling back with the catheter tip directed ventrally, and the catheter tip was placed proximal to the celiac artery for angiography (Figure 3A). A 1.7-Fr microcatheter (1.7-Fr microcatheter, Estream 1.7, TORAY, Tokyo, Japan) was inserted through the 4.2-Fr RIM-S guiding catheter. Referring to the blood vessel running obtained by the angiography (Figure 3A), the common hepatic artery was isolated, the microcatheter was advanced to the front of this branch, and angiography was performed to identify tumor feeding vessels in the caudate lobe region. An angled 0.014-inch microguidewire (1.4-Fr microguidewire, Cross Winder, TORAY, Tokyo, Japan) was inserted, and the feeding vessel was advanced and traced with a microcatheter. Angiography of the vicinity of the tumor revealed that the entire tumor was stained intensely (Figure 3B).

Gelpart (Nippon Kayaku, Tokyo, Japan), composed of fine grains of gelatin sponge (1-mm grain), was used for hemostasis. Before use, the particle size of Gelpart was reduced by the following method. After injecting 10 mL of iohexol contrast medium (300 mgI/mL) into a vial containing Gelpart, mixing was performed by inversion; the mixture was aspirated into a 2.5-mL syringe, which was then connected to another empty 2.5-mL syringe and a three-way stopcock at a 90° angle. The particle size was reduced by reciprocating the inner cylinder of the syringe 20 times with the three-way stopcock open. We reconfirmed the presence of the microcatheter tip in the target vessel with a test injection of the contrast agent. After confirmation, the embolizing agent was slowly injected into the target vessel under fluoroscopy guidance. Care was taken to prevent the flow of the embolization agent into vessels other than the target vessel (Figure 3C). The infusion was terminated immediately before complete stasis of blood flow in the target vessel. The microcatheter was flushed with physiological saline to complete the procedure and flush out any remaining particles in the microcatheter. Angiography was performed from the microcatheter to confirm occlusion of the target blood vessel; then, the microcatheter, 4.2-Fr RIM-S guiding catheter, and sheath were removed, and the puncture site of the femoral artery and the skin of the inguinal region was sutured. The total procedure time was 80 min (excluding the time for CT).

### Postoperative Course

**Figure 4:**
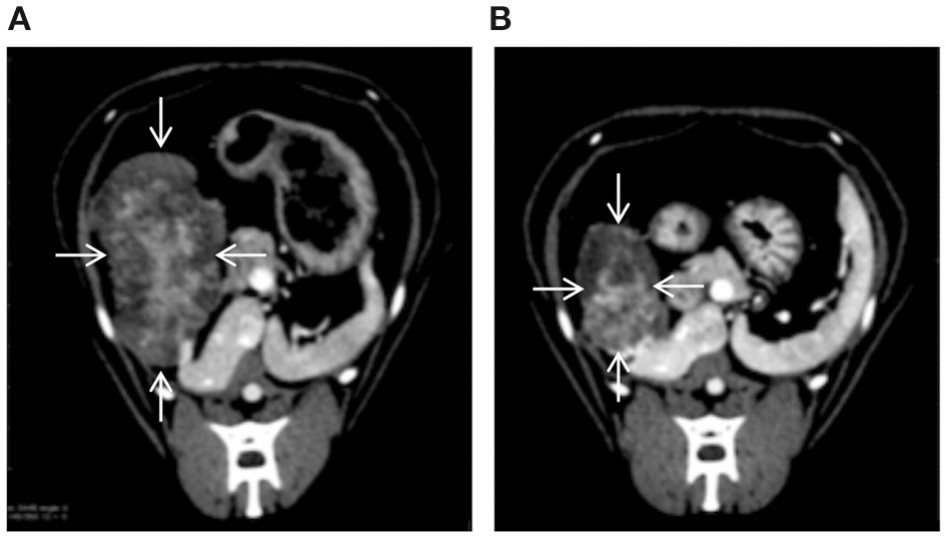
CT images showing that TAE reduced the size of the hepatic tumor (arrows) [**(A)**: before TAE, **(B)**: 66 days after TAE].

On day 1 after TAE, the cat's body temperature increased to 39.5°C, and the serum alanine aminotransferase (ALT) level increased to 303 U/L (reference range, 22–84 U/L). Therefore, lactated Ringer's solution was infused intravenously (3.0 mL/kg/h). On day 3 after TAE, the body temperature decreased to 38.6°C, and administration of lactated Ringer's solution was stopped. The cat was discharged 5 days after TAE. The ALT level, which was at its highest on postoperative day 1, gradually decreased from day 2 and eventually normalized to 66 U/L on day 11 after TAE. Moreover, there was no significant increase in alkaline phosphatase levels after embolization. Abdominal ultrasonography showed gradual disappearance of fluid in the abdominal cavity, and there was no fluid in the peritoneal cavity at discharge. At the time of discharge, the general condition of the cat was stable, both vigor and appetite were good„ and PCV had increased to 25%. After discharge, the cat's condition remained stable, and no further intra-abdominal bleeding occurred. The remaining mass was surgically removed 66 days after TAE. CT performed before resection revealed a reduction in the mass's longest diameter from 32 × 45 × 52 mm to 22 × 43 × 32 mm (a partial response) (Figure 4). Histopathological examination of the resected mass revealed a cholangiocellular adenoma. The cat was doing well at the last follow-up visit on day 549 after TAE.

## Discussion

In a previous study on dogs, approximately 70% of non-traumatic intra-abdominal hemorrhages were attributable to mass lesions, which are most likely to occur in the spleen, liver, adrenal glands, kidneys, intra-abdominal blood vessels, and abdominal wall (3, 4). Most mass lesions if managed promptly have a good prognosis. In that study, 41% of dogs with mass lesions survived and were discharged (4).

In a previous study in cats, only 46% (30/65) of non-traumatic hemorrhage cases were a result of neoplasia. The spleen was the most common location of neoplasia (11/30; 37%), followed by the liver (9/30; 30%). Moreover, only eight of the 65 cats in that study survived, and the prognosis for intra-abdominal hemorrhage was considerably worse in cats than in dogs (5).

After a canine or feline patient's hemodynamics are stabilized, laparotomy is generally performed to maintain hemostasis (3, 4). In humans, TAE is the first-line choice to maintain hemostasis for intra-abdominal hemorrhages caused by rupture of a hepatic mass when patients' hemodynamics are already stable (6). This approach is preferred because treatment results are better when TAE is used to control bleeding and stabilize the patient before mass removal rather than performing an emergency laparotomy to control the bleeding.

In this study, TAE was performed on a cat with a hepatic mass accompanied by intra-abdominal hemorrhage. The cat's condition stabilized, and the tumor size was reduced, and surgical resection to prevent rebleeding was possible with good general condition. Arterial embolization procedures are now performed in veterinary practice for liver tumors that cannot be surgically removed, with the aim of palliative mass reduction (7–9). Arterial embolization combined with arterial infusion chemotherapy is commonly performed for the same purpose in our hospital. This strategy appears to be effective for the initial management of intra-abdominal hemorrhage caused by tumors, as in the present case. In this case, the embolization procedure was completed in 80 min (excluding the time for CT); this duration was relatively short compared to that for traditional surgery, and this approach was considered minimally invasive.

In this case, identification of the bleeding site (extrahepatic extravasation) could not be confirmed by preoperative CT examination. However, bloody ascites was observed around the tumor, and the tumor was stained more intensely in the arterial phase than in the portal phase. Even in human medicine, it has been reported that extravasation is only observed on angiography in 13.2–35.7% of patients (10). In many cases, the site of bleeding must be inferred from the presence of a protruding tumor on the liver surface and the amount of blood flow. In recent years, the utility of preoperative CT has been investigated in identifying the bleeding site in small animal medical care (11). In the future, with the availability of more sophisticated equipment, preoperative CT may enable more detailed identification of the bleeding site, and more precise hemostasis by endovascular treatment may be possible.

In human medicine, platinum microcoils, polyvinyl alcohol or acrylic polymeric beads, and gelatin sponges are used as embolic materials for hemostasis of bleeding hepatic tumors (6). In the absence of controlled studies, there is currently no consensus as to which material is optimal for embolization. In this case, the diameter of the first branch of the feeding artery was 0.6 mm, and we used gelatin sponge, for which the particle size can be adjusted. Gelpart is a commercialized gelatin sponge approved for use in TAE. It is used in human TAE after reducing its particle size by crushing with a three-way stopcock and two syringes. It is said that pumping the syringe 20 times will result in a particle diameter of 0.5 mm and performing this process 50 times will result in a diameter of 0.2 mm (12). Since the diameter of the feeding artery branching into the tumor was 0.6 mm at the time of TAE, Gelpart was used after pumping the syringe 20 times. We injected it into the feeding artery while looking at the fluoroscopic image until the arterial blood flow began to stagnate and finally flushed the microcatheter with saline.

A previous study reported complications of TAE in a cat owing to the sharp angle between the aorta and celiac artery in cats (9). They used a carotid artery approach to prevent this complication. Therefore, we used a RIM-type guiding catheter, which matched the angle between the aorta and celiac axis. The RIM catheter has a hook shape and is used to select the celiac artery and superior mesenteric artery in human medicine (13). It enabled us to approach the celiac artery from the femoral artery. The femoral artery approach should be selected if possible, as placing the sheath in the femoral artery has the advantage of being less surgically invasive than placing the sheath in the carotid artery.

In this case, a fever of 39.5°C and an increase in the ALT level were observed on the 1st day after TAE, but treatment with intravenous fluids improved the cat's condition in approximately 72 h. The symptoms are similar to those of post-embolization syndrome in human medicine, and it is likely that the cat developed a corresponding condition. Post-embolization syndrome is a potential complication of TAE that can be caused by tumor cell necrosis. This complication is particularly common with a sufficient embolus, and it is caused by tumor necrosis (14). Major symptoms include fever, elevated ALT and aspartate transaminase levels, nausea, vomiting, and abdominal pain, though most cases resolve within a few days of symptom onset and with symptomatic treatment if necessary (15).

The limitation of this report is that extravasation was not confirmed as described above. Based on the circumstantial evidence, the peritoneal fluid accumulation was assumed to be a hemorrhage from the hepatic mass, and treatment was performed accordingly.

In our case, we performed TAE for the initial management of intra-abdominal hemorrhage from a hepatic mass in a cat. The condition of the cat was stabilized, and no further intra-abdominal bleeding occurred. The tumor was reduced in size, and surgical resection could be performed in good general condition. In human medicine, it is common to stop bleeding from an intra-abdominal tumor using TAE, stabilize the patient's condition, and then perform surgical resection. In veterinary medicine, it may be possible to improve the outcome of bleeding from an intra-abdominal mass by first stopping bleeding with TAE and then performing surgery, if necessary, after the animal's condition has stabilized. We would like to further investigate the effectiveness of TAE in treating intra-abdominal hemorrhage in cats.

## Data Availability Statement

The original contributions presented in the study are included in the article/supplementary material, further inquiries can be directed to the corresponding author/s.

## Ethics Statement

Ethical review and approval was not required for the animal study because this is a case report of a single cat that received veterinary diagnostics and treatments as clinically indicated. Written informed consent was obtained from the owner for the participation of their animal in this study.

## Author Contributions

YK, HI, AK, HS, and KK helped with the diagnosis and participated in clinical case management. YK participated in the review and editing of the manuscript. All authors contributed to the article and approved the submitted version.

## Conflict of Interest

The authors declare that the research was conducted in the absence of any commercial or financial relationships that could be construed as a potential conflict of interest.

## Publisher's Note

All claims expressed in this article are solely those of the authors and do not necessarily represent those of their affiliated organizations, or those of the publisher, the editors and the reviewers. Any product that may be evaluated in this article, or claim that may be made by its manufacturer, is not guaranteed or endorsed by the publisher.

## Abbreviations

ALT: Alanine aminotransferase
CT: computed tomography
HU: Hounsfield unit
IV: intravenous
PCV: packed cell volume
TAE: transcatheter arterial embolization.
